# Older Adults, the “Social Admission,” and Nonspecific Complaints in the Emergency Department: Protocol for a Scoping Review

**DOI:** 10.2196/38246

**Published:** 2023-03-15

**Authors:** Kayla Rose Furlong, Kathleen O'Donnell, Alison Farrell, Susan Mercer, Paul Norman, Michael Parsons, Christopher Patey

**Affiliations:** 1 Discipline of Family Medicine Faculty of Medicine Memorial University St John’s, NL Canada; 2 Discipline of Emergency Medicine Faculty of Medicine Memorial University St John's, NL Canada; 3 Carbonear Institute for Rural Reach and Innovation by the Sea Carbonear, NL Canada; 4 Health Sciences Library Memorial University Libraries Memorial University St John's, NL Canada

**Keywords:** social admission, failure to thrive, failure to cope, acopia, community emergency, geriatric emergency, emergency medicine, geriatric, older adults, elderly, hospital, visit, admission, non-medical, non-acute, social, reasons

## Abstract

**Background:**

Older adults have a higher visit rate and poorer health outcomes in the emergency department (ED) compared to their younger counterparts. Older adults are more likely to require additional resources and hospital admission. The nonspecific, atypical, and complex nature of disease presentation in older adults challenges current ED triage systems. Acute illness in older adults is often missed or commonly disguised in the ED as a social or functional issue. If diagnostic clarity is lacking or safe discharge from the ED is not feasible, then older adults may be labelled a “social admission” (or another synonymous term), often leading to negative health consequences.

**Objective:**

This scoping review aims to describe and synthesize the available evidence on patient characteristics, adverse events, and health outcomes for older adults labelled as “social admission” (and other synonymously used terms), as well as those with nonacute or nonspecific complaints in the ED or hospital setting.

**Methods:**

A literature search of MEDLINE, Embase, Scopus, PsycINFO, and CINAHL was completed. Relevant reference lists were screened. Data have been managed using EndNote software and the Covidence web application. Original data have been included if patients are aged ≥65 years and are considered a “social admission” (or other synonymously used term) or if they present to the ED with a nonacute or nonspecific complaint. Two review team members have reviewed titles and abstracts and will review full-text articles. Disagreements are resolved by consensus or in discussion with a third reviewer. This review does not require research ethics approval.

**Results:**

As of January 2023, we have completed the title and abstract screening and have started the full-text screening. Some remaining full-text articles are being retrieved and/or translated. We are extracting data from included studies. Data will be presented in a narrative and descriptive manner, summarizing key concepts, patient characteristics, and health outcomes of patients labelled as a “social admission” (and other synonymously used terms) and of those with nonacute and nonspecific complaints. We expect the first results for publication in Spring 2023.

**Conclusions:**

Acute illness in the older adult is not always easily identified. We hope to better understand patient characteristics, adverse events, and health outcomes of older adults labelled as a “social admission,” as well as those with nonacute or nonspecific complaints. We aim to identify priorities for future research and identify knowledge gaps that may inform health care providers caring for these vulnerable patients.

**International Registered Report Identifier (IRRID):**

DERR1-10.2196/38246

## Introduction

### Background

In recent years, visits to the emergency department (ED) by older adults (generally those aged ≥65 years) have significantly increased [1-3]. Between 2021 and 2050, the global population of older adults is expected to double [4], likely exacerbating the already existing problem of ED overcrowding [5]. The ED often serves as a “point of entry” for older adults, particularly for those without access—or perceived lack of access—to primary care [6,7]. Compared to younger counterparts, older adults in the ED have a higher visit rate and a longer length of stay and are more likely to be admitted to the hospital [3,8,9]. Older adults who visit the ED have a high mortality rate and increased likelihood of dependence for instrumental and basic activities of daily living [1,2].

The nonspecific and atypical nature of disease presentation in older adults presents a challenge in the ED. For example, issues may be missed at triage (ie, undertriaged), as many triage tools do not identify atypical, nonspecific, or multisystem complaints commonly seen in older adults [10-12]. Furthermore, older adults may present with no apparent acute medical reason for their visit, or at least none that can be quickly identified [13,14]. However, it is known that older adults who present with nonspecific complaints, such as “fatigue,” “weakness,” or “feeling unwell,” are at risk for serious conditions (ie, acute coronary syndrome, metabolic or endocrine disturbances, transient ischemic attack, urinary tract infection, dehydration, etc) [15,16]. Older adults may also present with a combination of acute, chronic, social, or functional issues [17-20]. If diagnostic clarity is lacking or if patients are unable to be safely discharged—but they also do not seemingly require a hospital bed—they are often labelled as a “social admission” [17,19]. Other synonymous labels include “failure to cope/thrive” [13,21]; “community emergencies” [22]; or other colloquial terms based on local hospital policy, culture, or media [19,21]. There is no single common term used to describe this older adult population. However, it is known that these patients are at high risk for inappropriate disposition and adverse outcomes [23].

Older adults typically present with geriatric syndromes, including delirium, falls, poorly managed pain, malnutrition, depression, functional decline, sensory impairment, and incontinence [17,19]. If older adults present with poorly controlled or multiple geriatric syndromes, then a “social admission” may occur if no specific diagnosis can be identified [21]. Not infrequently, these patients cannot return to their prior place of residence without additional support, which may require extensive discharge liaison and planning. Examples of these discharge predicaments include the need for additional home supports (including nursing and/or social supports), home equipment or structural remodeling to accommodate physical decline, or placement in a care facility, among others.

Many of these older patients present to the ED in the later stages of their complex health issues and may not return home [21]. There are a few potential contributing factors that may lead to a “social admission,” which include patient (eg, multimorbidity, polypharmacy, frailty, medication adverse events, or progression of or poorly managed health conditions), family/informal caregiver (eg, caregiver burnout, lack of a “back-up” plan, or time and financial constraints for caregiver), peer group (ie, social isolation), institutional (eg, single-system complaints or barriers to formal caregivers), or societal and policy (eg, government policy, accessibility, or age-friendly activities) factors [17,24-29]. If admitted, many health care providers may continue to view these patients as “bed blockers” or “inappropriate admissions” [19,28]. In addition, community-based health care professionals lack the resources, collaborative networks, and organizational infrastructure necessary to provide medical, social, and functional support outside of the hospital setting [30-32]. Thus, these patients continue to use the ED [33,34] and are at risk for further decline (eg, pressure ulcers, sleep deprivation, or delirium) once there [2,35-37]. Several studies also report high mortality rates among older adults with “acopia” (22%) [13], “failure to thrive” (32.5%) [38], and nonspecific chief complaints (20.2%) [39] that present to the ED.

Ideally, alternative pathways to care should exist outside the hospital setting, but our health care systems have not adapted to the demographic changes and increased health care needs of older adults [40]. Thus, many EDs remain ill-equipped to provide adequate care for these patients [40,41]. Historically, medical education systems train physicians and health care systems are designed with a focus on single-system complaints. However, this approach does not effectively meet the needs of older adults with nonacute, nonspecific, or multifactorial complaints [19,21]. The issue is perpetuated when ED health care professionals lack the expertise or comfort in managing these complex patients, while ED trainees often prefer to focus their efforts on more acute presentations [19]. ED-trained health care professionals are not equipped to manage these patient presentations from a time, training, or resource perspective [19,21].

While many EDs have implemented programs or interventions for older adults, they do not specifically target the older adults that present as a “social admission,” “failure to cope/thrive,” or “community emergency” [22,42]. There is a paucity of data around the patient characteristics, adverse events, and health outcomes of these patients that present to the ED. In this scoping review, we hope to describe what is currently known about these patients and identify knowledge gaps that may direct future research or interventions to potentially improve health outcomes.

### Review Aim and Objectives

The aim of this scoping review is to describe older adults (aged ≥65 years) who present to the ED and are labelled as a “social admission” (or other synonymous terms) or present with nonspecific complaints or without an apparent acute medical reason for their visit. The review objective is to summarize patient characteristics, adverse events, exposures, interventions, and disposition from the ED/hospital from the included study population.

## Methods

### Overview

The protocol was developed based on the proposed framework from the Joanna Briggs Institute for scoping reviews [43], which builds on the framework previously described by Arksey and O’Malley [44]. The PRISMA-ScR (Preferred Reporting Items for Systematic Reviews and Meta-Analyses Extension for Scoping Reviews) checklist has guided the reporting of this protocol and will guide the future reporting of the review itself [45] (see Multimedia Appendix 1).

A scoping review was felt to be the most appropriate to (1) map key concepts and evidence available, (2) summarize current evidence, and (3) identify knowledge gaps in the literature. We also recognize the need for a scoping review to be iterative and that the protocol may deviate to obtain the best results. If a study author identifies the need for a protocol update, other authors will be notified and involved in the process. A consensus will be reached between authors. Protocol deviations will be reported to *JMIR Research Protocols*.

### Inclusion and Exclusion Criteria

The inclusion and exclusion criteria are described in Table 1. The criteria are not exhaustive and may be modified throughout the search process, as necessary. If a study author identifies a search term, type of participant, variable, or outcome that they deem potentially relevant, other authors will be notified. A consensus will be reached between authors on whether to update the inclusion and exclusion criteria.

**Table 1:** Inclusion and exclusion criteria.

Criteria	Description
Types of participants	Inclusion criteria All patients who present to the emergency department (ED) without an apparent acute medical reason for their visit or a combination of acute, chronic, functional, or social issues (ie, nonacute or nonspecific complaints) Labelled as a “social admission” or other synonymous term (ie, failure to thrive/cope, community emergency, etc) or with clinically relevant presentation Male or female Aged ≥65 years Exclusion criteria Aged <65 years Not labelled as a “social admission” or other synonymous term or with nonacute or nonspecific complaints
Variables and outcomes	We will collect data, as available, to include: All types of patient characteristics^a^ All exposures in the ED/hopsital^a^ All adverse events in the ED/hospital^a^ All interventions in the ED/hospital^a^ Disposition from the ED/hospital^a^ All other measured outcomes reported in included studies^b^
Setting	ED Hospital
Types of evidence sources	Original journal articles (no systematic reviews or meta-analyses) Observational studies (cohort, case control, or cross-sectional), descriptive studies, case series, case reports, opinion pieces, and letters to the editor^c^

^a^See Textbox 1 for details.

^b^When an outcome is identified by an author as being potentially relevant, that author will notify the other authors. All authors involved in the title and abstract review, full-text review, and data extraction processes will be informed, and a decision will be made via consensus.

^c^Any opinion piece or letter to the editor that contains original research data will be included.

**Variables for data extraction.**

- Publication details (ie, author, journal, year of publication, country, and grey or peer-reviewed)
- Methodological data (ie, study type, sample size, etc)
- Patient characteristics (ie, age, past medical history, presenting complaint, triage score, arrival mode, place of residence, recent admissions, recent emergency department visits, etc)
- Adverse events (ie, falls, delirium, pain, infection, etc)
- Exposures (ie, infectious diseases, antipsychotic medication use, etc)
- Interventions (ie, antibiotics, intravenous fluids, new medications, etc)
- Other measured health outcomes
- Disposition (ie, home, home with supports, nursing homes, long-term care, etc)

### Search Strategy

A research librarian, in collaboration with the review team, developed search strategies to identify potentially relevant articles for screening. The following databases will be searched: (1) MEDLINE, (2) Embase, (3) Scopus, (4) PsycINFO, and (5) CINAHL. The search strategies are presented in Multimedia Appendices 2-6. Search dates are from 1946 to November 8, 2022. Some of the search terms included are “social admission,” “social patient,” “acopia,” “bed blocker,” geriatric emergenc*,” and “community emergency,” among others (see Multimedia Appendix 1). The search strategies were piloted by ensuring that they captured a few key articles that are likely to be included in the final review. Records will be exported to EndNote software (Clarivate Analytics) [46] for deduplication and citation management and then into Covidence [47] for article screening. A final search will be repeated prior to the draft of the manuscript. Searches of the databases will be supplemented by manual searches through the references lists of original journal article publications, as well as citation searching when appropriate. If a relevant review or meta-analysis is identified, we will manually search the associated reference list.

### Study Selection Process

Two reviewers will independently screen titles and abstracts identified by all electronic databases against the predetermined inclusion and exclusion criteria (see Table 1) and document their findings in Covidence [47]. To ensure interrater reliability, 10% of the titles and abstracts were piloted before starting the formal screening process. A flow chart will be used to report the study selection process according to the PRISMA-ScR statement. Any disagreement of the titles and abstracts identified between reviewers will be resolved via consensus between reviewers or via arbitration by a third reviewer if consensus is not achievable.

Next, articles that are deemed relevant will be reviewed in full and screened according to the inclusion and exclusion criteria. Any disagreement of the full-text review between reviewers will be resolved via consensus between reviewers or via arbitration by a third reviewer if consensus is not achievable. When a duplicate study is identified, the data will be extracted from the most informative study sample, but all published articles will be included in the reference list. When important data are missing, the authors of all eligible studies will be contacted.

### Data Charting, Analysis, and Synthesis

Reviewers will use standardized pro forma data extraction Microsoft Excel sheets to independently extract relevant data from included studies. To ensure interrater reliability, we will pilot 10 studies prior to formally starting the data collection process using the data extraction sheets. Variables and data to be extracted are listed in Textbox 1. Other key information related to the review objectives may also be collected. Further data points may be added if unforeseen, useful data can be extracted. We will provide a narrative synthesis of study data. Text and tables will be used to provide a descriptive summary of included studies and study characteristics.

### Risk of Bias (Quality Assessment)

As this is a scoping review of the available evidence, no quality assessment will be performed.

## Results

Our search strategies were applied to the 5 databases as described above (see *Search Strategy*). Prior to starting data collection, 2 authors (KRF and KOD) piloted 100 studies to review their titles and abstracts. Disagreement was identified for 8 (8%) studies. All disagreements were resolved via consensus between the 2 reviewers (KRF and KOD). A third reviewer was not required.

In total, 1860 titles and abstracts were identified for screening after the removal of 115 duplicate articles. Thus far, 30 full-text articles were identified for inclusion. As of January 2023, we have completed the title and abstract screening. The full-text review is ongoing as we await the retrieval of some texts and translation of others. A manual reference search has been completed. We are currently extracting data from identified studies for inclusion. We will present data in a narrative and descriptive manner. We expect the first results for publication in Spring 2023.

## Discussion

### Expected Findings

The results of this scoping review aim to inform ED physicians and other ED health care practitioners about this population. We hope that appropriate stakeholders and policy makers can adapt their approach to the population labelled as a “social admission” and with nonacute, nonspecific complaints. We aim to describe the characteristics of this population, how they present, and any exposures or adverse events experienced during their ED stay or “social admission.” It is expected that this population may demonstrate frailty, comorbidity, and polypharmacy. We expect the reasons for presentation to include nonspecific complaints, such as “weakness” or “generally unwell,” but to also include functional or social reasons that are difficult for ED physicians to navigate. We expect acute medical illnesses to be missed and for disposition and discharge planning to be difficult. We also expect adverse events, revisit, admission, and mortality rates to be high, if documented.

To date, several original journal articles have been published in this area. However, the terms used to describe this population varies greatly between institutions and over decades. To our knowledge, this is the first review to be conducted on this topic with the intention of including all synonymous and colloquial terms in the literature equivalent to “social admission” and nonacute and nonspecific complaints in the ED. Strengths of our study include our broad-scope search strategies and the inclusion of a wide range of study designs. We chose to conduct a scoping review and not a systematic review as we wanted to focus on the state of research that exists for this emerging topic, not necessarily its quality. Limitations may include the inability to make evidence-based recommendations for clinical guidelines and a lack of risk of bias assessment for included full-text studies.

### Dissemination Plans

We hope that this scoping review identifies priority areas for future research. Dissemination will help practitioners and the public understand this patient population. A multidisciplinary approach will help guide the dissemination of results at local hospitals and clinical sites. We plan to publish our results in a peer-reviewed academic journal and present at national and international conferences.

## Appendix

Multimedia Appendix 1 PRISMA-ScR (Preferred Reporting Items for Systematic Reviews and Meta-Analyses Extension for Scoping Reviews) checklist.

Multimedia Appendix 2 MEDLINE search strategy.

Multimedia Appendix 3 Embase search strategy.

Multimedia Appendix 4 Scopus search strategy.

Multimedia Appendix 5 PsycINFO search strategy.

Multimedia Appendix 6 CINAHL search strategy.

## Abbreviations

ED: emergency department
PRISMA-ScR: Preferred Reporting Items for Systematic Reviews and Meta-Analyses Extension for Scoping Reviews

## Data Availability

The data sets generated during and/or analyzed during the current study will be available from the corresponding author on reasonable request.
