# Supplementary material for: Older Adults, the “Social Admission,” and Nonspecific Complaints in the Emergency Department: Protocol for a Scoping Review
Source: JMIR Res Protoc. 2023 Mar 15;12:e38246. doi: 10.2196/38246 (PMC10132007; doi:10.2196/38246)
Supplement: Multimedia Appendix 2 [file resprot_v12i1e38246_app2.docx]

Ovid MEDLINE(R) and Epub Ahead of Print, In-Process, In-Data-Review & Other Non-Indexed Citations and Daily <1946 to November 08, 2022>

| 1 | Failure to Thrive/ | 2395 |
| --- | --- | --- |
| 2 | ("community emergencies" or "community emergency" or "social admission*" or "non-operative injur*" or "non acute" or nonacute or "social patient*" or acopia or "lack of community support" or "bed blocker*" or "geriatric emergenc*" or "non specific complaint*" or "non-specific complaint*" or "vague symptom*" or "orphan patient*" or "home care impossible" or gomer or gomers or "get out of my emergency room" or "GP problem*" or "medically inappropriate" or "placement problem*").tw,kf. | 4101 |
| 3 | ((failure or fail or failing or inabilit* or "reduced abilit*" or unable) adj3 (cope or manage or thrive)).tw,kf. | 7866 |
| 4 | 1 OR 2 OR 3 | 12988 |
| 5 | exp Emergency Service, Hospital/ | 95973 |
| 6 | (emergency adj (room or department or service or services or ward or unit)).tw,kf. | 136063 |
| 7 | ER.tw,kf. | 104295 |
| 8 | 5 OR 6 OR 7 | 279021 |
| 9 | exp Aged/ | 3422616 |
| 10 | Geriatrics/ | 31185 |
| 11 | (aging or ageing or senior* or elder* or older or aged or old).tw,kf. | 2541990 |
| 12 | 9 OR 10 OR 11 | 5232414 |
| 13 | 4 AND 8 AND 12 | 493 |
| 14 | ("social admission*" or "non-operative injur*" or "social patient*" or acopia or "bed blocker*" or "geriatric emergenc*").ti. | 205 |
| 15 | 13 OR 14 | 573 |
