# Supplementary material for: Older Adults, the “Social Admission,” and Nonspecific Complaints in the Emergency Department: Protocol for a Scoping Review
Source: JMIR Res Protoc. 2023 Mar 15;12:e38246. doi: 10.2196/38246 (PMC10132007; doi:10.2196/38246)
Supplement: Multimedia Appendix 3 [file resprot_v12i1e38246_app3.docx]

Embase Search History

Run Nov 18, 2022

| 1 | **'failure to thrive'/exp** | 11472 |
| --- | --- | --- |
| 2 | **'community emergencies' OR 'community emergency' OR 'social admission*' OR 'non-operative injur*' OR 'non acute' OR nonacute OR 'social patient*' OR acopia OR 'bed blocker*' OR 'geriatric emergenc*' OR 'non specific complaint*' OR 'non-specific complaint*' OR 'vague symptom*' OR 'orphan patient' OR 'home care impossible' OR gomer OR gomers OR 'get out of my emergency room' OR 'gp problem*' OR 'medically inappropriate' OR 'placement problem*'** | 7985 |
| 3 | **(failure OR fail OR failing OR inability OR unable) NEAR/3 (cope OR manage OR thrive)** | 16906 |
| 4 | **#1 OR #2 OR #3** | 22304 |
| 5 | **'emergency ward'/exp** | 172727 |
| 6 | **emergency NEAR/1 (room OR department OR service OR services OR ward OR unit)** | 271358 |
| 7 | **Er: ti,ab** | 149777 |
| 8 | **#5 OR #6 OR #7** | 414447 |
| 9 | **'aged'/exp** | 3399328 |
| 10 | **aging:ti,ab OR ageing:ti,ab OR senior*:ti,ab OR elder*:ti,ab OR older:ti,ab OR aged:ti,ab OR old:ti,ab** | 3331932 |
| 11 | **‘geriatrics'/exp** | 55497 |
| 12 | **#9 OR #10 OR #11** | 5831891 |
| 13 | **#4 AND #8 AND #12** | 799 |
| 14 | **'social admission*':ti OR 'non-operative injur*':ti OR 'social patient*':ti OR acopia:ti OR 'bed blocker*':ti OR 'geriatric emergenc*':ti** | 298 |
| 15 | **#13 OR #14** | 933 |
