# Supplementary material for: Older Adults, the “Social Admission,” and Nonspecific Complaints in the Emergency Department: Protocol for a Scoping Review
Source: JMIR Res Protoc. 2023 Mar 15;12:e38246. doi: 10.2196/38246 (PMC10132007; doi:10.2196/38246)
Supplement: Multimedia Appendix 4 [file resprot_v12i1e38246_app4.docx]

Scopus Search

Updated Nov 18, 2022

( ( ( TITLE-ABS-KEY ( ( "community emergencies"  OR  "community emergency"  OR  "social admission*"  OR  "non-operative injur*"  OR  "non acute"  OR  nonacute  OR  "social patient*"  OR  acopia  OR  "bed blocker*"  OR  "geriatric emergenc*"  or “non specific complaint*” or “non-specific complaint*” or “vague symptom*” or “orphan patient” or “home care impossible” or gomer or gomers or “get out of my emergency room” or “GP problem*” or “medically inappropriate” or “placement program*”) ) )  OR  ( TITLE-ABS-KEY ( ( ( failure  OR  fail  OR  failing  OR  inability  OR  unable )  W/3  ( cope  OR  manage  OR  thrive ) ) ) ) )  AND  ( ( TITLE-ABS-KEY ( ( emergency  W/1  ( room  OR  department  OR  service  OR  services  OR  ward  OR  unit ) ) ) )  OR  ( TITLE-ABS-KEY ( er ) ) )  AND  ( TITLE-ABS-KEY ( ( aging  OR  ageing  OR  senior*  OR  elder*  OR  older  OR  aged  OR  old ) ) ) )  OR  ( TITLE ( ( "social admission*"  OR  "non-operative injur*"  OR  "social patient*"  OR  acopia  OR  "bed blocker*"  OR  "geriatric emergenc*" ) ) )
