# Supplementary material for: Older Adults, the “Social Admission,” and Nonspecific Complaints in the Emergency Department: Protocol for a Scoping Review
Source: JMIR Res Protoc. 2023 Mar 15;12:e38246. doi: 10.2196/38246 (PMC10132007; doi:10.2196/38246)
Supplement: Multimedia Appendix 5 [file resprot_v12i1e38246_app5.pdf]

EBSCOhost

Wednesday, November 23, 2022 3:22:31 PM

#	Query	Limiters/Expanders	Last Run Via	Results
S13	S11 OR S12	Expanders - Apply equivalent subjects<br>Search modes - Boolean/Phrase	Interface - EBSCOhost Research Databases<br>Search Screen - Advanced Search<br>Database - APA PsycInfo	47
S12	TI ("social admission*" or "non-operative injur*" or "social patient*" or acopia or "bed blocker*" or "geriatric emergenc*")	Expanders - Apply equivalent subjects<br>Search modes - Boolean/Phrase	Interface - EBSCOhost Research Databases<br>Search Screen - Advanced Search<br>Database - APA PsycInfo	16
S11	S3 AND S7 AND S10	Expanders - Apply equivalent subjects<br>Search modes - Boolean/Phrase	Interface - EBSCOhost Research Databases<br>Search Screen - Advanced Search<br>Database - APA PsycInfo	44
S10	S8 OR S9	Expanders - Apply equivalent subjects<br>Search modes - Boolean/Phrase	Interface - EBSCOhost Research Databases<br>Search Screen - Advanced Search<br>Database - APA PsycInfo	645,093
S9	TI ((aging or ageing or senior* or elder* or older or aged or old)) OR AB ((aging or ageing or senior* or elder* or older or aged or old))	Expanders - Apply equivalent subjects<br>Search modes - Boolean/Phrase	Interface - EBSCOhost Research Databases<br>Search Screen - Advanced Search<br>Database - APA PsycInfo	639,854
S8	DE "Older Adulthood" OR DE "Geriatric Patients" OR DE "Geriatrics" OR DE "Geriatric Assessment"	Expanders - Apply equivalent subjects<br>Search modes - Boolean/Phrase	Interface - EBSCOhost Research Databases<br>Search Screen - Advanced Search<br>Database - APA PsycInfo	43,588

S7	S4 OR S5 OR S6	Expanders - Apply equivalent subjects<br>Search modes - Boolean/Phrase	Interface - EBSCOhost Research Databases<br>Search Screen - Advanced Search<br>Database - APA PsycInfo	31,969
S6	ER	Expanders - Apply equivalent subjects<br>Search modes - Boolean/Phrase	Interface - EBSCOhost Research Databases<br>Search Screen - Advanced Search<br>Database - APA PsycInfo	7,001
S5	(emergency N1 (room or department or service or services or ward or unit))	Expanders - Apply equivalent subjects<br>Search modes - Boolean/Phrase	Interface - EBSCOhost Research Databases<br>Search Screen - Advanced Search<br>Database - APA PsycInfo	25,582
S4	DE "Emergency Services"	Expanders - Apply equivalent subjects<br>Search modes - Boolean/Phrase	Interface - EBSCOhost Research Databases<br>Search Screen - Advanced Search<br>Database - APA PsycInfo	9,982
S3	S1 OR S2	Expanders - Apply equivalent subjects<br>Search modes - Boolean/Phrase	Interface - EBSCOhost Research Databases<br>Search Screen - Advanced Search<br>Database - APA PsycInfo	6,064
S2	((failure or fail or failing or inabilit* OR "reduced abilit*" or unable) N3 (cope or manage or thrive))	Expanders - Apply equivalent subjects<br>Search modes - Boolean/Phrase	Interface - EBSCOhost Research Databases<br>Search Screen - Advanced Search<br>Database - APA PsycInfo	5,507
S1	("community emergencies" or "community emergency" or "social admission*" or "non-operative injur*" or "non acute" or nonacute or "social patient*" or acopia OR "lack of community support" or "bed	Expanders - Apply equivalent subjects<br>Search modes - Boolean/Phrase	Interface - EBSCOhost Research Databases<br>Search Screen - Advanced Search<br>Database - APA PsycInfo	558

blocker*" or "geriatric emergenc*" or "non specific complaint*" or "non-specific complaint*" or "vague symptom*")
