# Supplementary material for: Older Adults, the “Social Admission,” and Nonspecific Complaints in the Emergency Department: Protocol for a Scoping Review
Source: JMIR Res Protoc. 2023 Mar 15;12:e38246. doi: 10.2196/38246 (PMC10132007; doi:10.2196/38246)
Supplement: Multimedia Appendix 6 [file resprot_v12i1e38246_app6.pdf]

Thursday, November 17, 2022 2:06:23 PM

#	Query	Limiters/Expanders	Last Run Via	Results
S14	S12 OR S13	Expanders - Apply equivalent subjects<br>Search modes - Boolean/Phrase	Interface - EBSCOhost Research Databases<br>Search Screen - Advanced Search<br>Database - CINAHL Plus with Full Text	404
S13	TI ("social admission*" or "non-operative injur*" or "social patient*"or acopia or "bed blocker*" or "geriatric emergenc*")	Expanders - Apply equivalent subjects<br>Search modes - Boolean/Phrase	Interface - EBSCOhost Research Databases<br>Search Screen - Advanced Search<br>Database - CINAHL Plus with Full Text	184
S12	S7 AND S8 AND S11	Expanders - Apply equivalent subjects<br>Search modes - Boolean/Phrase	Interface - EBSCOhost Research Databases<br>Search Screen - Advanced Search<br>Database - CINAHL Plus with Full Text	320
S11	S9 OR S10	Expanders - Apply equivalent subjects<br>Search modes - Boolean/Phrase	Interface - EBSCOhost Research Databases<br>Search Screen - Advanced Search<br>Database - CINAHL Plus with Full Text	1,324,954
S10	(aging or ageing or senior* or elder* or older or aged or old)	Expanders - Apply equivalent subjects<br>Search modes - Boolean/Phrase	Interface - EBSCOhost Research Databases<br>Search Screen - Advanced Search<br>Database - CINAHL Plus with Full Text	1,322,591
S9	(MH "Aged+") OR (MH "Geriatrics")	Expanders - Apply equivalent subjects<br>Search modes - Boolean/Phrase	Interface - EBSCOhost Research Databases<br>Search Screen - Advanced Search<br>Database - CINAHL Plus with Full Text	940,605

S8	S4 OR S5 OR S6	Expanders - Apply equivalent subjects<br>Search modes - Boolean/Phrase	Interface - EBSCOhost Research Databases<br>Search Screen - Advanced Search<br>Database - CINAHL Plus with Full Text	161,463
S7	S1 OR S2 OR S3	Expanders - Apply equivalent subjects<br>Search modes - Boolean/Phrase	Interface - EBSCOhost Research Databases<br>Search Screen - Advanced Search<br>Database - CINAHL Plus with Full Text	5,888
S6	ER	Expanders - Apply equivalent subjects<br>Search modes - Boolean/Phrase	Interface - EBSCOhost Research Databases<br>Search Screen - Advanced Search<br>Database - CINAHL Plus with Full Text	55,835
S5	(emergency N1 (room or department or service or services or ward or unit)	Expanders - Apply equivalent subjects<br>Search modes - Boolean/Phrase	Interface - EBSCOhost Research Databases<br>Search Screen - Advanced Search<br>Database - CINAHL Plus with Full Text	136,593
S4	(MH "Emergency Service+")	Expanders - Apply equivalent subjects<br>Search modes - Boolean/Phrase	Interface - EBSCOhost Research Databases<br>Search Screen - Advanced Search<br>Database - CINAHL Plus with Full Text	70,677
S3	((failure or fail or failing or inability or "reduced abilit*" or unable) N3 (cope or manage or thrive))	Expanders - Apply equivalent subjects<br>Search modes - Boolean/Phrase	Interface - EBSCOhost Research Databases<br>Search Screen - Advanced Search<br>Database - CINAHL Plus with Full Text	2,619
S2	("community emergencies" or "community emergency" or "social admission*" or "lack of community support" or "non-operative injur*" or "non acute" or nonacute or "social patient*" or acopia or "bed blocker*"	Expanders - Apply equivalent subjects<br>Search modes - Boolean/Phrase	Interface - EBSCOhost Research Databases<br>Search Screen - Advanced Search<br>Database - CINAHL Plus with Full Text	1,797

	or "geriatric emergenc*" or "nonspecific complaint*" or "non-specific complaint*" or "vague symptom*" or "orphan patient" OR "home care impossible" OR gomer OR gomers OR "get out of my emergency room" or "GP problem*" OR medically inappropriate" OR "placement problem*"))			
S1	(MH "Failure to Thrive") OR (MH "Failure to Diagnose")	Expanders - Apply equivalent subjects<br>Search modes - Boolean/Phrase	Interface - EBSCOhost Research Databases<br>Search Screen - Advanced Search<br>Database - CINAHL Plus with Full Text	2,420
